# Pseudohexagonal Nb_2_O_5_ Anodes for Fast-Charging Potassium-Ion Batteries

**DOI:** 10.1021/acsami.2c21490

**Published:** 2023-03-21

**Authors:** Guanxu Chen, Jintao Chen, Siyu Zhao, Guanjie He, Thomas S. Miller

**Affiliations:** Electrochemical Innovation Lab, Department of Chemical Engineering, UCL, London WC1E 7JE, U.K.

**Keywords:** KIB, potassium-ion batteries, carbon nanomaterials, PIB, niobium oxide, battery anode

## Abstract

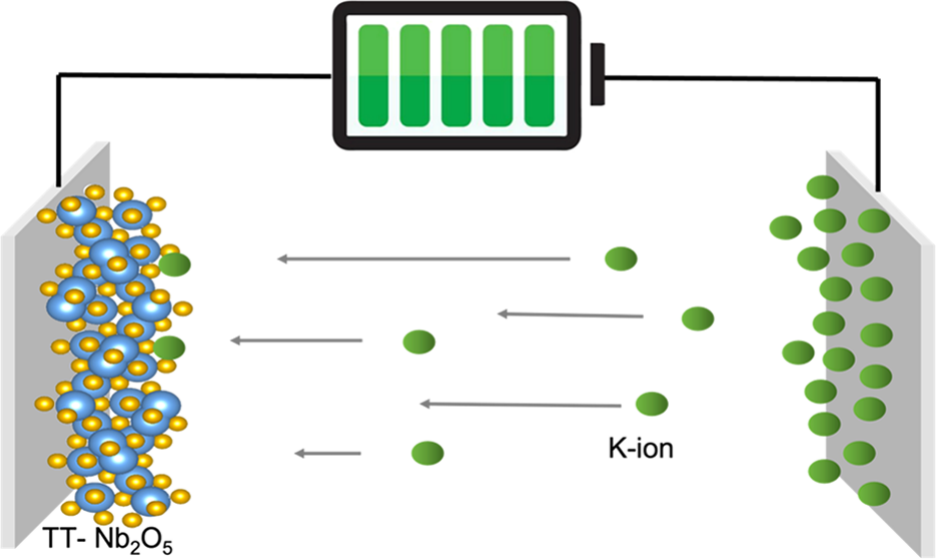

High-rate batteries will play a vital role in future energy storage systems, yet while good progress is being made in the development of high-rate lithium-ion batteries, there is less progress with post-lithium-ion chemistry. In this study, we demonstrate that pseudohexagonal Nb_2_O_5_(TT-Nb_2_O_5_) can offer a high specific capacity (179 mAh g^–1^ ∼ 0.3C), good lifetime, and an excellent rate performance (72 mAh g^–1^ at ∼15C) in potassium-ion batteries (KIBs), when it is composited with a highly conductive carbon framework; this is the first reported investigation of TT-Nb_2_O_5_ for KIBs. Specifically, multiwalled carbon nanotubes are strongly tethered to Nb_2_O_5_ via glucose-derived carbon (Nb_2_O_5_@CNT) by a one-step hydrothermal method, which results in highly conductive and porous needle-like structures. This work therefore offers a route for the scalable production of a viable KIB anode material and hence improves the feasibility of fast-charging KIBs for future applications.

## 1 Introduction

The growing demand for electric vehicles and portable electronic devices is driving the need for energy storage systems that overcome the numerous disadvantages associated with current technologies.^1^ For example, while lithium-ion batteries (LIBs) exhibit a reasonable capacity and reliable electrochemical performance, the limited availability and uneven global distribution of lithium^2^ means that their future application is at risk of over-demand or sociopolitical changes.^4^ Potassium-ion batteries (KIBs) are promising alternatives as, among other factors, K has a low standard electrode potential (Li/Li^+^: −3.04 V vs standard hydrogen electrode (SHE), K/K^+^: −2.93 V vs SHE) contributing to a large potential window and a high energy density.^3^ However, unlike lithium, potassium is relatively abundant (>2% of the earth’s crust composition)^4^ and much more equitably distributed around the globe, offering significant benefits in terms of resource security and sustainability.^5−7^ Unfortunately, despite KIBs generally relying on similar intercalation, alloying, or conversion reactions similar to those used in LIBs, materials for K storage often suffer from low capacities, short lifetimes, or poor rate capabilities due to the significantly larger ionic radius of K^+^ (1.38 vs 0.76 Å for Li^+^).^8−10^

Achieving a high charge–discharge rate is a key challenge for KIBs, as K ions exhibit poor kinetics due to their large radius, and materials that have been used to try to achieve the high performance can largely be divided into two main categories: carbonaceous (e.g., hard–soft carbon composites,^11^ N–S co-doped carbons,^12^ phosphorus-doped carbons^13^) and noncarbonaceous (e.g., SnP_2_O_7_ composites,^14^ potassium tetratitanate,^15^ or azobenzene-4,4′-dicarboxylic acid potassium salts—ADAPTS^16^). Yet unfortunately, carbonaceous anodes can achieve good cyclability, but they exhibit relatively poor rate compatibility at scan rates higher than 1C. Regarding the pure noncarbonaceous materials, the high cost of raw materials hindered their commercialization. In addition, noncarbonaceous anodes, in general, cannot achieve good cyclability, good rate performance at scan rate over 20C, and high specific capacity at the same time.

Niobium (V) oxide (Nb_2_O_5_) has been widely investigated as a high-rate anode for LIBs as it offers cyclic stability, high capacity, and outstanding reversibility.^17−21^ It has also gained attention as an anode for sodium-ion batteries (SIBs) in recent years, despite its somewhat modest rate performance.^22−29^ However, to date, only a small number of studies have examined the electrochemical performance of Nb_2_O_5_ as an anode material for KIBs, despite the fact it has a large lattice spacing (3.93 Å) that can accommodate K ions.^30^ Nonetheless, previous reports have shown that the orthorhombic form of Nb_2_O_5_(T-Nb_2_O_5_) can offer good capacities in KIBs at low rates and over reasonably long cycle lifetimes.^24−27^ For example, Li et al.^31^ investigated T-Nb_2_O_5_ nanowires as KIB anodes, which delivered a specific capacity of 127 mAh g^–1^ at 0.2 A g^–1^, whereas Yuan et al.^32^ designed a T-Nb_2_O_5_ N-doped carbon composite, which showed a capacity of 161 mAh g^–1^ at 0.1 A g^–1^. However, they also show that T-Nb_2_O_5_ in KIBs does not perform as well at high rates as it does in LIBs. Nonetheless, other forms of Nb_2_O_5_ do exist, such as pseudohexagonal Nb_2_O_5_(TT-Nb_2_O_5_), which offers benefits including lower-temperature synthetic conditions and a tendency toward a smaller average particle size.^33^ Yet, TT-Nb_2_O_5_ has not, to date, been assessed as an anode material for KIBs.

Like many other transition metal oxides (TMOs), Nb_2_O_5_ is a semiconductor with a relatively wide band gap, meaning that it can be difficult to use directly as an electrode in most batteries due to its low electrical conductivity.^34,35^ The two most widely applied strategies to overcome this issue are either reducing the size of the TMOs to the nanoscale or combining the TMOs with conductive materials (often carbonaceous—e.g., graphite,^18,36^ graphene,^37−39^ reduced graphene oxide (rGO),^40,41^ or carbon nanotubes (CNTs)^42−45^). CNTs, as commercialized and cost-effective materials, offer particular advantages in electrode composites as their excellent mechanical, electrical, and thermal properties will increase electrical conductivity, enhance electrode stability, and improve the electrode structure. However, to uniformly deposit TMO nanoparticles (NPs) onto CNTs is challenging as unfunctionalized CNTs are difficult to disperse evenly in aqueous solutions and tend to form weak links with nanoparticles due to the lack of surface groups to act as sites for tethering.^41^

Herein, we assess the capability of pseudohexagonal Nb_2_O_5_ for use as anodes in KIBs for the first time, enabled via a designed carbon framework. Although TT-Nb_2_O_5_ is less ordered than its crystalline analogue (T-Nb_2_O_5_), it can be synthesized in less extreme conditions, which favors nanostructuring and scale-up.^39,46,47^ By synthetically combining TT-Nb_2_O_5_ and high-conductivity multiwalled carbon nanotubes (MWCNTs), strongly bound together with a glucose-derived carbon to form a well-integrated composite electrode, the conductivity, mechanical strength, stability, and specific capacity of the anode material were optimized. The needle-like Nb_2_O_5_@CNT composites have delivered a high reversible specific capacity of 170 mAh g^–1^ at 0.2 A g^–1^ and excellent rate retention when compared to T-Nb_2_O_5_-based KIB systems to date: 102 mAh g^–1^ at 2 A g^–1^ and 72 mAh g^–1^ at 5 A g^–1^. This therefore offers a scalable solution to the development of sustainable high-rate energy storage systems.

## 2 Methodology

### 2.1 Synthesis of Nb_2_O_5_/g-CNT Composites

Nb_2_O_5_@CNT was synthesized using a one-step hydrothermal method followed by annealing. 0.4 g of ammonium niobate oxalate hydrate, 35 mg of multiwalled carbon nanotubes (outer diameter × inner diameter × length = 10 ± 1 nm × 4.5 ± 0.5 nm × 3 to ∼6 μm; Sigma-Aldrich, U.K.), and 0.4 g of glucose (Sigma-Aldrich, U.K.) were uniformly mixed and ground with a mortar and pestle before they were dispersed into 30 mL of methanol/DI water (1:1 in volume ratio). The homogeneous suspension was transferred to a Teflon-lined autoclave (4744 Acid Digestion Vessel, Parr) and heated in an oven at 180 °C for 12 h while the hydrothermal reaction proceeded. The as-synthesized powder was washed with DI water and freeze-dried (PowerDry LL3000, Thermo Scientific, U.K.) before being annealed at 500 °C for 3 h in an argon atmosphere using a tube furnace. The same methodology was applied for the synthesis of pure Nb_2_O_5_ without the addition of MWCNTs and glucose. Similarly, the glucose-derived carbon/MWCNT composite (g-CNT) was synthesized by mixing MWCNT and glucose as above and treating them under the same hydrothermal and annealing conditions.

### 2.2 Material Characterization

The crystal structure and phase of the as-received powder samples were analyzed by powder X-ray diffraction (XRD, STOE SEIFERT, Cu foil mode). The surface chemistry of the samples was analyzed by X-ray photoelectron spectroscopy (XPS, Thermo-Scientific, K-alpha photoelectron spectrometer), and the data were processed using CASA XPS with calibration of the binding energy position with C 1s at 284.8 eV. The surface area and pore size of the composite were analyzed by N_2_ adsorption–desorption measurements (Micromeritics, Flex&3Flex). The morphology of the Nb_2_O_5_@CNT powder was analyzed by scanning electron microscopy (SEM, JOEL JSM6701 FEG-SEM), and the chemical structure of the materials was further analyzed by Raman spectroscopy (laser wavelength of ∼540 nm, DXR3 Raman Microscope). Elemental analysis of the composite was conducted by energy-dispersive spectroscopy (EDS, Oxford INCA X-act). The bulk composition of each sample was determined by thermogravimetric analysis (TGA, Mettler Toledo, TGA/DSC 3+).

### 2.3 Electrochemical Characterization

Nb_2_O_5_@CNT and Nb_2_O_5_ anodes were prepared by mixing homogeneous slurries of the active material, carbon black (Sigma-Aldrich, U.K.), and poly(vinylidene fluoride) (PVDF, MTI, U.K.) in *N*-methyl-2-pyrrolidoone (NMP, Sigma-Aldrich U.K.) in a weight ratio of 7:2:1 using a Thinky mixer (Thinky ARE-250). The slurry was then coated onto a 25 μm thick aluminum current collector via doctor blading. After drying in a vacuum oven (Binder, U.K.) for 12 h at 120 °C, the electrodes were cut to a diameter of 16 mm and assembled into CR2032 coin cells in an argon-filled glovebox at room temperature (∼25 °C) with a glass fiber separator (Whatman GF/D), 90 μL of potassium bis(fluorsulfonyl)imide (KFSI, Combi-Blocks, US) in ethylene carbonate (EC)/diethyl carbonate (DEC) (1:1 in volume ratio, Sigma-Aldrich, U.K.) as the electrolyte, and potassium foil (Sigma-Aldrich, U.K.) as the counter electrode. Cyclic voltammetry (CV) tests were conducted using the Biologic battery cycler (Biologic BCS-800) within 0.01 and 3 V (vs K/K^+^) at scan rates between 0.1 and 2 mV s^–1^. The charge–discharge (CD) performances of Nb_2_O_5_@CNT and Nb_2_O_5_ were also analyzed using a battery cycler in the potential window of 0.01–3.0 V (vs K/K^+^) at current densities between 0.1 and 5 A g^–1^. Electrochemical impedance spectroscopy (EIS) was conducted using a Biologic potentiostat (Biologic VSP-300) with an amplitude of 10 mV in the frequency range of 100 kHz to 0.1 mHz.

## 3 Results and Discussion

**Figure 1:**
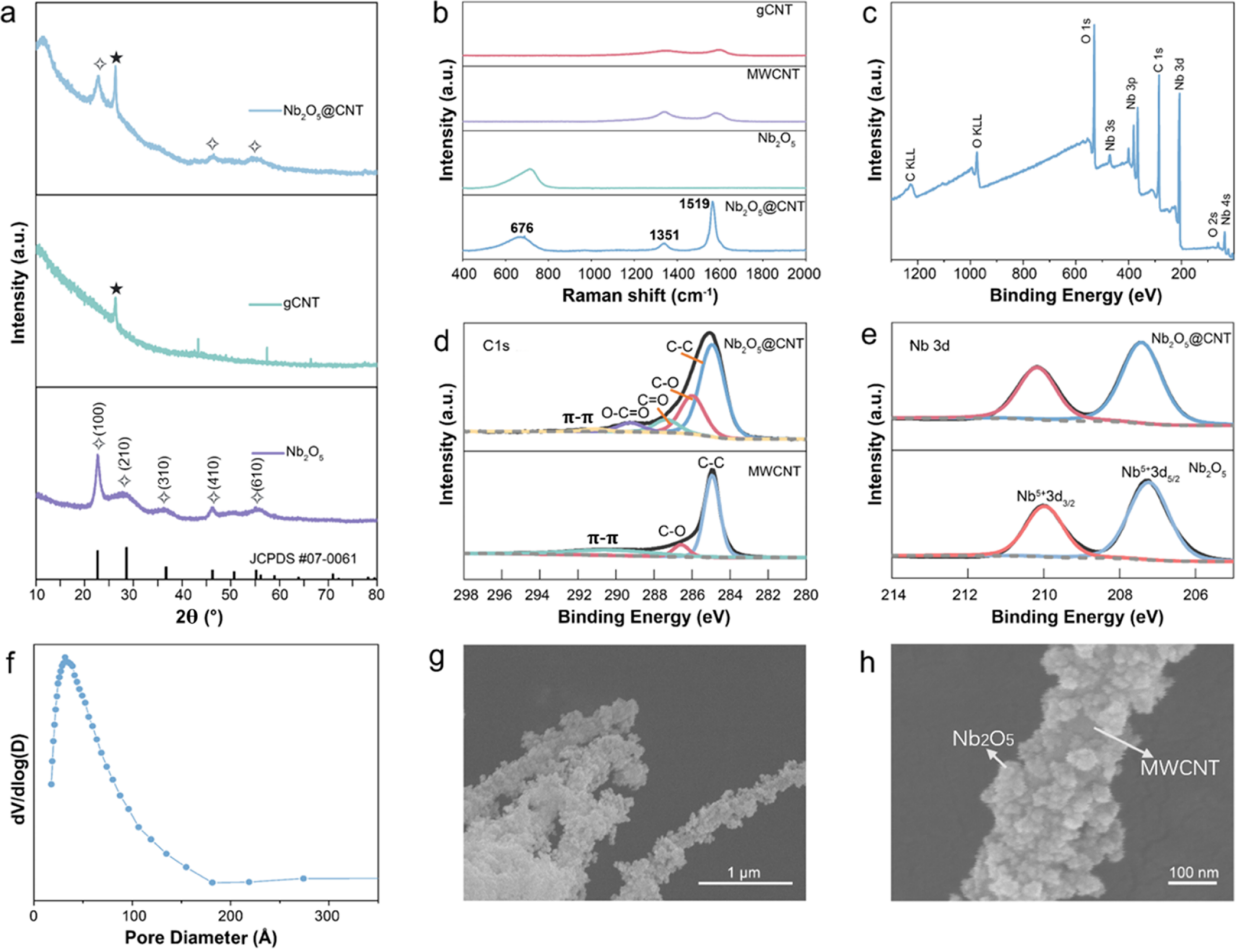
(a) XRD patterns of Nb_2_O_5_@CNT, Nb_2_O_5_, and gCNT. (b) Raman spectra of Nb_2_O_5_@CNT, Nb_2_O_5_, MWCNT, and gCNT. (c) XPS survey spectrum of Nb_2_O_5_@CNT. (d) C 1s XPS spectra of Nb_2_O_5_@CNT and MWCNT. (e) Nb 3d spectra of Nb_2_O_5_@CNT and Nb_2_O_5_. (f) Pore size distribution of Nb_2_O_5_@CNT. (g, h) SEM images of Nb_2_O_5_@CNT.

Pseudohexagonal Nb_2_O_5_, glucose-derived-carbon-decorated multiwalled carbon nanotubes (gCNT), and their composites (Nb_2_O_5_@CNT) were synthesized via a one-step hydrothermal method and post-annealing (see Figure S1, Supporting Information). The XRD patterns of Nb_2_O_5_@CNT, gCNT, and Nb_2_O_5_ are shown in Figure 1a. Characteristic peaks for the (100), (210), (310), (400), and (610) reflections can be observed for Nb_2_O_5_, which can be assigned to the pseudohexagonal form of Nb_2_O_5_ (TT- Nb_2_O_5_, JCPDS #07-0061). TT-Nb_2_O_5_ has a crystal structure similar to that of T-Nb_2_O_5_, but it consists of distorted sub-cells with a pseudohexagonal symmetry in a superlattice.^39,46^ The diffraction pattern of gCNT shows the typical (002) plane of CNTs at 2θ ≈ 26°, and the Nb_2_O_5_@CNT pattern can be interpreted as a combination of the contributions from TT-Nb_2_O_5_ and gCNT.^48^ The particle size of Nb_2_O_5_ within Nb_2_O_5_@CNT was estimated by the Scherrer equation (see eq S1) to be ∼8.47 nm. As the amorphous-glucose-derived carbon could not be detected via XRD, the overall composition of Nb_2_O_5_@CNT was analyzed by thermogravimetric analysis (TGA) (see Figure S2, Supporting Information). The mass loss of 3.21% at 230 °C can be attributed to the removal of absorbed water. The further losses of 21.33 and 21.32% at 261 and 571 °C can be attributed to the combustion of amorphous carbon and MWCNTs in the sample, respectively, leaving a 54.14% remainder due to residual Nb_2_O_5_. Hence, the overall Nb_2_O_5_-to-carbon ratio in the Nb_2_O_5_@CNT composite was close to 1.3:1.

Raman spectroscopy of Nb_2_O_5_@CNT was then performed to provide insights into the nature of the amorphous glucose-derived carbon (Figure 1b). The peaks centered at 676, 1351, and 1519 cm^–1^ correspond to vibrations of the Nb–O bonds, sp^3^ carbons (D-band), and graphitic carbons (G-band), respectively.^41,49−51^ In contrast to the Raman spectrum of pristine MWCNT, the intensity of graphitic carbon (G-band) is significantly higher than that of the disordered carbon (D-band) for Nb_2_O_5_@CNT, which may suggest that the hydrothermal treatment reduced the defect density in the framework of MWCNTs.^52^ Interestingly, this effect was not observed for gCNT, which was prepared using an equivalent hydrothermal method. This phenomenon may be caused by the surface-enhanced Raman scattering (SERS)-active performance of Nb_2_O_5_. Nb_2_O_5_ is a semiconductor with a high refraction index of 2.31, which means that the Mie resonance-generated light can be easily excited by Nb_2_O_5_ and lead to enhanced surface electric and magnetic fields.^53^ In addition, TT-Nb_2_O_5_ nanoparticles in the Nb_2_O_5_@CNT composite have rich surface active sites (Lewis sites and Brønsted sites), which could promote the interaction between Nb_2_O_5_ and the probe molecules on its surface and result in the enlarged Raman scattering cross section.^53,54^ The Raman spectras were further processed by peak simulation fitting to study the features of the graphite structure and to analyze defects within the carbon materials (see Figure S3, Supporting Information; D and G in the figure refer to the D and G peaks, respectively, which usually appear near 1350 and 1560 cm^–1^).^55^ By comparing the intensity ratio of the D- and G-bands, the structural perfection can be studied.^56,57^ The *I*_D_/*I*_G_ values of different materials were calculated to evaluate the defects of the structure and domain size of the carbon materials. The *I*_D_/*I*_G_ values of gCNT, MWCNT, and Nb_2_O_5_@CNT are 2.68, 1.69, and 0.17 accordingly, which indicates that the defect concentration was decreased after hydrothermal synthesis with the addition of Nb_2_O_5_. This, together with the Raman results, further confirmed the coexistence of Nb_2_O_5_, glucose-derived amorphous carbon, and MWCNTs and confirmed the high purity of the composite.

X-ray photoelectron spectroscopy (XPS) was used to analyze the surface chemistry of the samples. The XPS survey spectrum of Nb_2_O_5_@CNT (Figure 1c) shows no peaks for elements other than Nb, C, and O, as expected, further confirming its high purity. Comparisons of the Nb 3d and C 1s regions of Nb_2_O_5_@CNT, Nb_2_O_5_, and MWCNT are shown in Figure 1d,e. Nb^5+^ 3d_5/2_ and Nb^5+^ 3d_3/2_ peaks for Nb_2_O_5_@CNT can be found at 207.44 and 210.18 eV, which show no obvious change when compared to pure Nb_2_O_5_. This result illustrated that the hydrothermal reaction with the addition of MWCNT and glucose did not change the chemical state of Nb_2_O_5_.

Regarding the carbon contribution, the peak at 284.9 eV for MWCNT C 1s, which corresponds to the C–C bond in the carbon skeleton, was found to be broader in the C 1s of Nb_2_O_5_@CNT due to the addition of glucose-derived carbon. Besides this, peaks that can be assigned to C–O, C=O, and O–C=C bonds at 285.98, 287.34, and 289.23 eV can also be seen, which suggest that the glucose-derived carbon in the composite contains surface functional groups^55^ that may provide binding sites for Nb_2_O_5_ to anchor to the carbon framework. The atomic percentages of components of different chemical states are shown in Table SI. In the fitted C 1s spectrum of Nb_2_O_5_@CNT, 59.59 atom % of the C–C bonds (peak at 284.93 eV) are attributed to the bonding between MWCNT and glucose-derived carbon, whereas 22.74 atom % C–O, 8.31 atom % C=O, and 5.84 atom % O–C=O bonds are related to the functional groups of glucose-derived carbon.

The surface area and pore distribution of the Nb_2_O_5_@CNT composite were analyzed by N_2_ adsorption/desorption measurements at 77K (Figure S4, Supporting Information). The result shows a Brunauer–Emmett–Teller (BET) surface area of 519.75 m^2^ g^–1^ and a Barrett–Joyner–Halenda (BJH) average pore diameter of 64.57 Å (Figure 1f). The high surface area of the composite was achieved due to the combination of nanosized particles and their even distribution on CNTs. The N_2_ adsorption/desorption isotherm confirmed that the composite is mesoporous.^58^ The morphology of Nb_2_O_5_@CNT was further investigated by scanning electron microscopy (SEM; Figures 1g,h and S5, Supporting Information), which showed a needle-like structure consisting of Nb_2_O_5_ NPs uniformly coated on supporting MWCNTs, further confirmed by EDS (Figure S6, Supporting Information). Moreover, uneven distribution of Nb_2_O_5_ can be found in the SEM image of the Nb_2_O_5_@CNT composite with low carbon content (Figure S7, Supporting Information), which indicates that controlling the carbon content is extremely important in achieving a uniform structure of the composite. This high-surface-area nanostructure should provide enhanced electrical conductivity, good structural flexibility, and fast mass transport, thereby maximizing the capacity, rate performance, and stability of the composite when utilized as a KIB anode.

**Figure 2:**
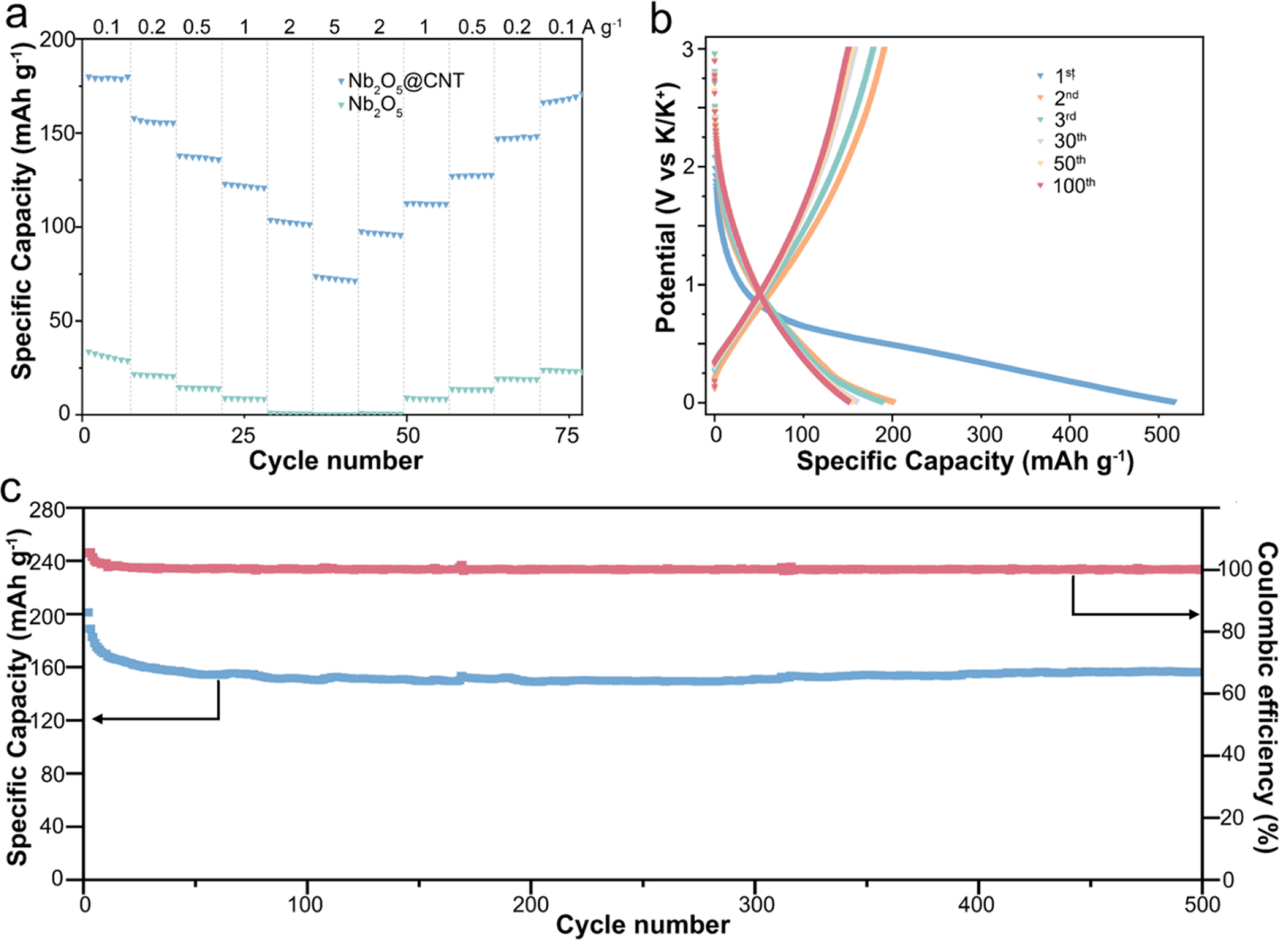
(a) Rate performances of Nb_2_O_5_@CNT (0.92 mg cm^–2^) and Nb_2_O_5_ (0.98 mg cm^–2^) at increasing current densities of 0.1, 0.2, 0.5, 1, 2, and 5 A g^–1^. (b) Charge/discharge performance of Nb_2_O_5_@CNT (1.03 mg cm^–2^) in the 1st, 2nd, 3rd, 30th, 50th, and 100th cycles at 0.2 A g^–1^. (c) Cycling performance of Nb_2_O_5_@CNT (1.03 mg cm^–2^) at 0.2 A g^–1^.

Figure 2 shows the electrochemical performance of Nb_2_O_5_@CNT as the active anode material in a KIB half-cell. The theoretical capacity of Nb_2_O_5_@CNT is 387 mAh g^–1^. The rate performance of Nb_2_O_5_@CNT and Nb_2_O_5_ are compared in Figure 2a; Nb_2_O_5_@CNT delivers capacities of 179, 156, 137, 121, 102, and 72 mAh g^–1^ at current densities of 0.1, 0.2, 0.5, 1, 2, and 5 A g^–1^ (from ∼0.3C to ∼15C) respectively, showing ∼5 times higher capacity compared to that of pure Nb_2_O_5_ at low rates during long cycles (see Figure S8, Supporting Information) and an order of magnitude higher capacities at high rates. The performance of Nb_2_O_5_@CNT at high rates is better than those of all Nb_2_O_5_-based KIB anodes in the literature to date (see Figure S9, Supporting Information). Moreover, these capacities are recoverable when the current density is decreased stepwise from 5 to 0.1 A g^–1^.

To understand the impact of the hydrothermally prepared carbon framework for TT-Nb_2_O_5_, the rate performance of Nb_2_O_5_@CNT with half the Nb_2_O_5_ content (0.2Nb_2_O_5_@CNT) and a simple mixture of MWCNT and TT-Nb_2_O_5_ was also tested (see Figure S10, Supporting Information). The 0.2Nb_2_O_5_@CNT delivered specific capacities of 98, 68, 42, 24, 5, and 0.3 mAh g^–1^ at current densities of 0.1, 0.2, 0.5, 1, 2, and 5 A g^–1^, indicating that the capacity of Nb_2_O_5_@CNT is mainly bestowed by TT-Nb_2_O_5_ (see Figure S11, Supporting Information). The mixture of MWCNT and TT-Nb_2_O_5_ delivered specific capacities of only 31, 21, 14, 9, 1, and 0.1 mAh g^–1^ as the current density was increased from 0.1 to 5 A g^–1^, showing that the glucose-derived carbon plays a vital role in achieving the capacity of the composite. The intimately linked structure is expected to significantly improve electrical conductivity and mass transport while offering mechanical stabilization.

Similar to other transition metal oxides, Nb_2_O_5_@CNT exhibited a relatively low first-cycle Coulombic efficiency (FCCE) of 38.9% (Figure 2b), which can be attributed to the formation of a solid electrolyte interface (SEI). However, after the first cycle, the change in the CD profile was minimal, which indicates good electrode and SEI stability at Nb_2_O_5_@CNT. In fact, the long-term cycling performance at a current density of 0.2 A g^–1^ (Figure 2c) shows that the specific capacity of the electrode is maintained at ∼152 mAh g^–1^ for a minimum of 500 cycles with Coulombic efficiencies close to 100%. A similar lifetime study was also conducted for glucose-coated MWCNTs to evaluate the impact of the carbon base on the composite capacity and stability. As shown in Figure S7, glucose-coated MWCNTs provide a specific capacity of 67 mAh g^–1^, whereas a simple Nb_2_O_5_ electrode exhibits a specific capacity of 12 mAh g^–1^ at 0.2 A g^–1^, demonstrating that the designed composite offers significant benefits over its constituent parts.

**Figure 3:**
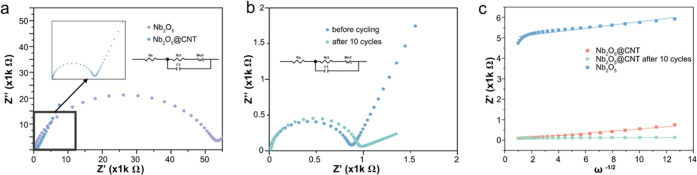
EIS plots of (a) Nb_2_O_5_@CNT and Nb_2_O_5_ and (b) Nb_2_O_5_@CNT before cycling and after 10 cycles. (c) Randles plot of the electrodes of Nb_2_O_5,_ Nb_2_O_5_@CNT, and Nb_2_O_5_@CNT after 10 cycles in the Warburg region.

Electrochemical impedance spectroscopy (EIS) measurements were conducted to confirm the differences in charge transfer and inherent resistance between Nb_2_O_5_@CNT and Nb_2_O_5_ (Figure 3a). Equivalent circuit models for both spectra are shown in Figure 3a,b, in which *R*_s_ and *R*_ct_ represent the ohmic resistance between the electrolyte/surface and charge-transfer resistance, respectively.^59^ The measured *R*_ct_ of Nb_2_O_5_@CNT was found to be 790 Ω, which is ∼66 times smaller than that of Nb_2_O_5_ (52 kΩ). The huge decrease in *R*_ct_ can be attributed to the addition of the MWCNT framework, which lowers the energy barrier for electron transfer in the composite. The Nyquist plot of Nb_2_O_5_@CNT before cycling was also compared with the equivalent after 10 cycles at 0.2 A g^–1^ (Figure 3b). The small increase in the diameter of the semicircle after 10 cycles can be attributed to the formation of an SEI film, which increases the charge-transfer resistance of the battery system. However, the magnitude is indicative of a compact and stable interface. The slope at low frequencies, corresponding to the Warburg impedance, is indicative of the K ion diffusion in the cell.^60−62^

To further study the capacitance behavior, the K-ion diffusion coefficient was calculated using the EIS data based on eq 1(63)
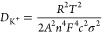
1where *D*_K^+^_ is the diffusion coefficient, *R* is the gas constant (8.314 J mol^–1^ K^–1^), *T* is the absolute temperature, *A* is the effective working area of the anode, *n* is the number of electrons transferred, *F* is the Faraday constant (96 500 C mol^–1^), *C* is the K concentration, and σ is the Warburg factor. The Warburg factor can be calculated by the linear Randles equation (eq 2)^64^
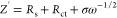
2

where ω in the angular frequency at low frequencies. As shown in Figure 3c, the slope of the simulated linear plots represents the Warburg factor (807.12, 536.88, and 31.38 Ω S^–1/2^ for Nb_2_O_5_ and Nb_2_O_5_@CNT before cycling and Nb_2_O_5_@CNT after 10 cycles, respectively). Consequently, the K-ion diffusion coefficients can be calculated to be 1.76 × 10^–14^, 3.97 × 10^–14^, and 1.16 × 10^–11^ cm^2^ s^–1^ for Nb_2_O_5_ and Nb_2_O_5_@CNT before cycling and Nb_2_O_5_@CNT after 10 cycles, respectively. The higher diffusion rate of Nb_2_O_5_@CNT after 10 cycles compared with the other two is because of the capacitance behavior of the anode before the exhibition of electrochemical reaction after SEI formation, which justifies its outstanding rate performance.^15^

**Figure 4:**
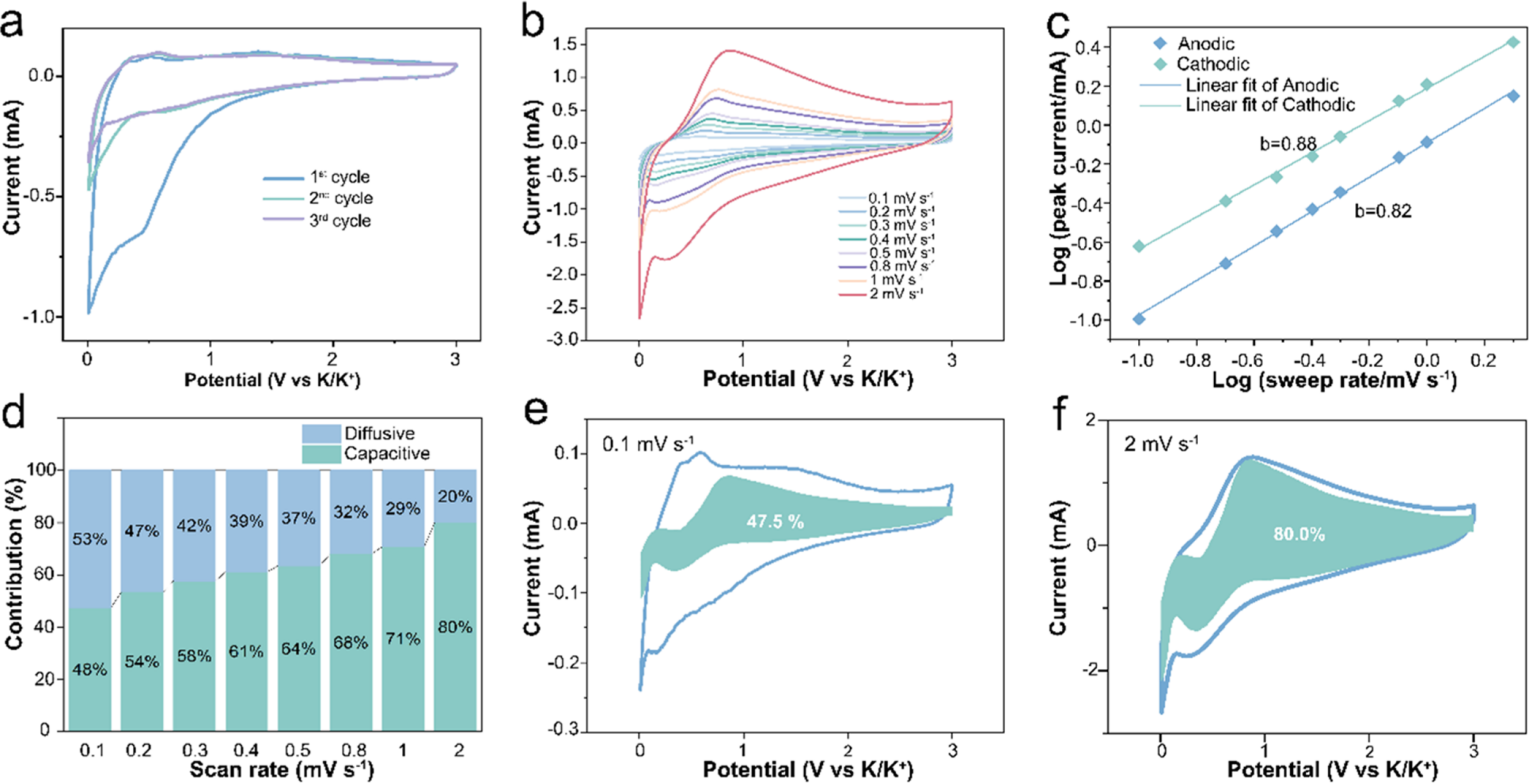
(a) First three CV cycles of Nb_2_O_5_@CNT at 0.1 mV s^–1^. (b) CV profiles of Nb_2_O_5_@CNT at different scan rates from 0.1 to 2 mV s^–1^. (c) Logarithmic relationship between the peak current and scan rates. (d) Contribution ratio of capacitive and diffusive reactions to the capacity of Nb_2_O_5_@CNT at different scan rates. Fitting of the pseudocapacitive contribution at (e) 0.1 and (f) 2 mV s^–1^.

To uncover the charge storage mechanism of Nb_2_O_5_@CNT in KIBs, cyclic voltammetry (CV) tests were conducted. The initial three CV cycles at 0.1 mV s^–1^ are shown in Figure 4a. The broad peak centered at 0.45 V, which is the dominant feature of the first cathodic scan, can be assigned to the formation of the SEI layer, caused by the irreversible decomposition of the KFSI-based electrolyte in the first cycle;^65^ this peak disappears by the 3rd cycle, which is important as it indicates the formation of a stable SEI. Interestingly, at low scan rates, few other faradic peaks can be resolved, but as shown in Figure 4b, the CV profile for Nb_2_O_5_@CNT changed as the scan rate was increased from 0.1 to 2 mV s^–1^. Two broad peaks, which correspond to the reaction Nb_2_O_5_ + *x*K^+^ ⇌ K*_x_*Nb_2_O_5_, become increasingly resolvable at higher scan rates while the distance between anodic and cathodic peaks increases, indicating an increase in the degree of polarization. To further reveal the mechanism of the potassiation/de-potassiation, the ex-situ XPS profile of cycled Nb_2_O_5_@CNT was studied. As the SEI layer formed on the surface of the cycled nanostructured anode, most of the contribution from the underlying electrodes was blocked. However, the mechanism can still be studied by comparing the changes in peak shifts at charge/discharge states in the XPS spectrum. As shown in Figure S12a,b, the decrease in the intensity of the C–C bond in the C 1s fitting is coupled with an increase in the intensities of K 2p peaks after discharge, which indicates the intercalation of K-ions and the formation of potassium–carbon compounds.^66^ The Nb 3d spectra indicates the reversible oxidation and reduction of Nb species in the fully charged and discharged states (see Figure SI12c,d, Supporting Information). The change in the intensity of Nb^5+^, which corresponds to the change in the intensity of Nb–O in the O 1s spectra, indicates that Nb^5+^ is reduced to lower valence states after discharge.^67^

Figure 4c shows the logarithmic relationship between the peak current of the cathodic and anodic processes (*i*) and scan rate (*v*), from which the capacitive contribution of Nb_2_O_5_@CNT can be determined by the power law^68,69^
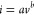
3where *a* and *b* are variable parameters. The *b*-value, which is the slope of the linear fit (Figure 4c), can be used to determine the Faradic and non-Faradic behaviors in the electrochemical process of Nb_2_O_5_@CNT. When the *b*-value is equal to 0.5, the electrochemical process is regarded as being controlled by a diffusion-controlled behavior, but if the *b*-value is close to 1, it is assumed to be dominated by a pseudocapacitive behavior.^70^ The *b*-values of Nb_2_O_5_@CNT were calculated to be 0.88 and 0.82 for the anodic and cathodic peaks, respectively, suggesting primarily pseudocapacitive storage in Nb_2_O_5_@CNT-based KIBs, indicating its fast kinetics. The contributions of diffusive and capacitive behaviors to the total capacity can be further analyzed quantitatively using eq 4(71)
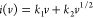
4where (*k*_1_*v*) represents the capacitive contribution and (*k*_2_*v*^1/2^) signifies the diffusive dominance. This calculation reveals that 48, 54, 56, 61, 64, 68, 71, and 80% of the capacity of Nb_2_O_5_@CNT were provided by (pseudo)capacitive reactions at scan rates of 0.1, 0.2, 0.3, 0.4, 0.5, 0.8, 1.0, and 2.0 mV s^–1^, respectively (Figure 4d). The capacitive contribution ratio increases with the increasing scan rate (Figure 4e,f), consistent with the high rate capability of Nb_2_O_5_@CNT, indicating that the high accessibility of the electrically connected Nb_2_O_5_ can lead to very fast redox reactions.

## 4 Conclusions

In summary, a composite of needle-like pseudohexagonal Nb_2_O_5_ on MWCNT was synthesized by a scalable hydrothermal method and studied as an anode for KIBs. Nb_2_O_5_@CNT exhibited a highly reversible specific capacity (179 mAh g^–1^) and excellent cyclability both in terms of long cycles (152 mAh g^–1^ at 0.2 A g^–1^ after 500 cycles) and at high rates (72 mAh g^–1^ at 0.5 A g^–1^). All of these metrics are the best for all reported Nb_2_O_5_-based KIB anodes to date. The enhanced electrochemical performance is attributed to the optimization of electron/ion diffusion kinetics, electrical conductivity, and mechanical stability offered by the composite. Importantly, this work therefore offers a scalable route for the production of a high-activity post-lithium-ion battery anode material, with significant implications for next-generation energy storage systems. With optimization, the material demonstrated could enhance the commercial viability of KIBs.

## Supporting Information Available

Schematic of the formation of the Nb_2_O_5_@CNT composite (Figure S1); TGA curve of Nb_2_O_5_@CNT (Figure S2); atomic percentage calculated by processing the fitted Nb 3d, C 1s, and O 1s XPS spectra (Table S1); Raman spectra of Nb_2_O_5_@CNT, Nb_2_O_5_ and gCNT (Figure S3); N_2_ adsorption/desorption isotherm of Nb_2_O_5_@CNT (Figure S4); TEM image of the Nb_2_O_5_@CNT composite (Figure S5); EDS profile of the Nb_2_O_5_@CNT composite (Figure S6); SEM image of Nb_2_O_5_@0.5CNT (Figure S7); CD profiles of Nb_2_O_5_@CNT (0.94 mg cm^–2^), g-MWCNT (0.81 mg cm^–2^), and Nb_2_O_5_ (0.96 mg cm^–2^) at 0.2 A g^–1^ (Figure S8); Comparison of Nb_2_O_5_@CNT composite in this paper with other reported Nb_2_O_5-_based KIB anode (Figure S9); Rate performance of pure Nb_2_O_5_ and mixture of Nb_2_O_5_ and MWCNT at different current densities (Figure S10); rate performance of 0.4Nb_2_O_5_@CNT (low carbon content) and 0.2Nb_2_O_5_@CNT (high carbon content) in KIBs (Figure S11) (PDF)
