# Supplementary material for: Pseudohexagonal Nb2O5 Anodes for Fast-Charging Potassium-Ion Batteries
Source: ACS Appl Mater Interfaces. 2023 Mar 21;15(13):16664–72. doi: 10.1021/acsami.2c21490 (PMC10080539; doi:10.1021/acsami.2c21490)
Supplement: Supplementary file 1 — am2c21490_si_001.pdf [file am2c21490_si_001.pdf]

SUPPORTING INFORMATION

Pseudohexagonal Nb_2O_5 Anodes for Fast-Charging Potassium-Ion Batteries

Guanxu Chen[a], Jintao Chen[a], Siyu Zhao[a], Guanjie He[a*] and Thomas S. Miller[a*].

[a]Electrochemical Innovation Lab, Department of Chemical Engineering, UCL, London, WC1E 7JE, UK
* Corresponding author: T.Miller@ucl.ac.uk, g.he@ucl.ac.uk

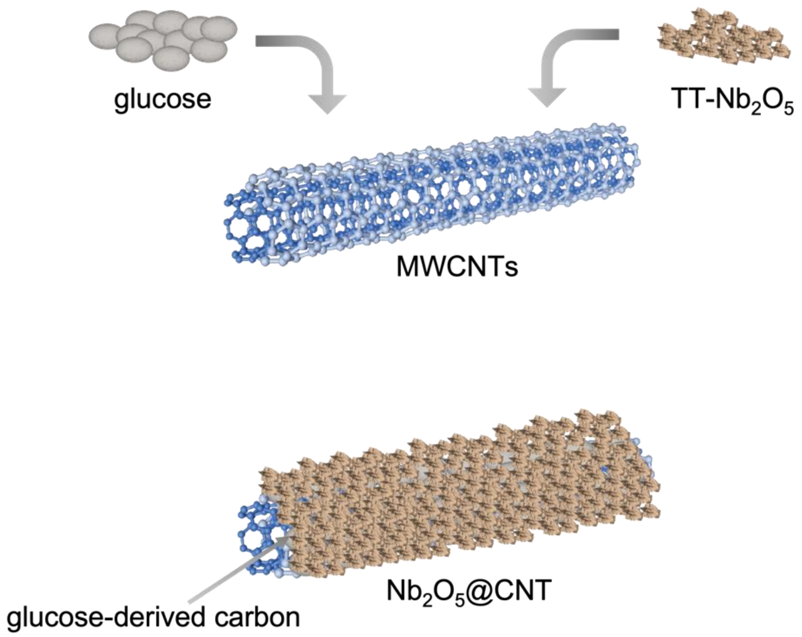

Figure S1 Schematic of the formation of the Nb_2O_5@CNT composite.

Scherrer equation:

$$d = \frac{0.9\lambda}{\beta \cos\theta} \qquad \text{Eq.S1}$$

where d is the size of the particle, λ is the wavelength of the X-ray, β is FWHM and θ is the diffraction angle.

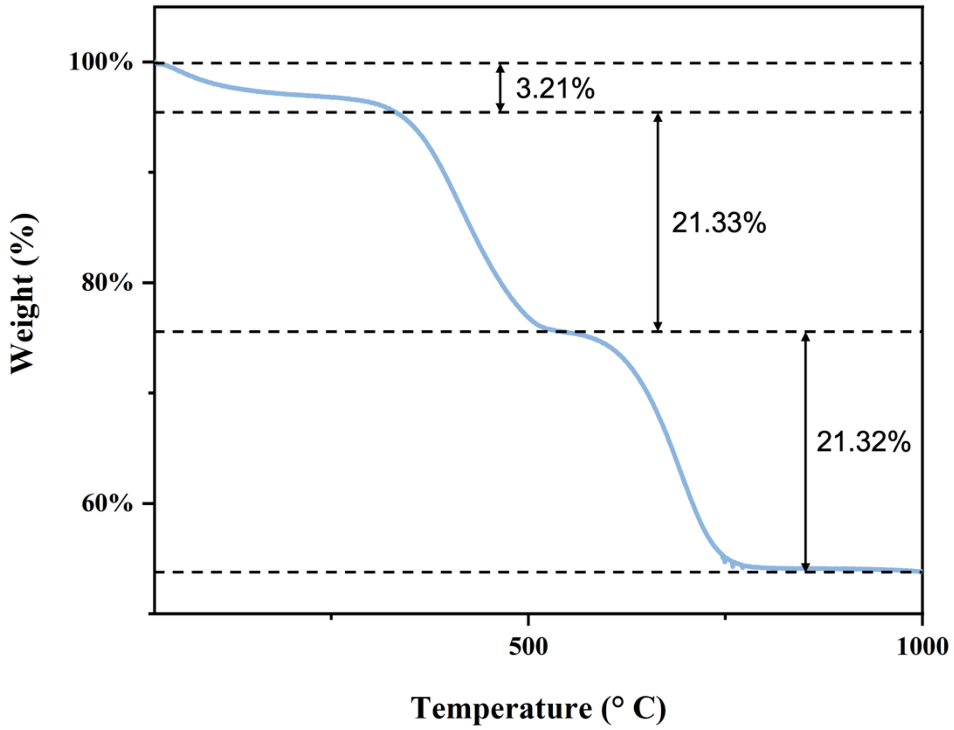

Figure S2 TGA curve of the Nb_2O_5@CNT.

The TGA curve of Nb_2O_5@CNT is as shown in Figure S1. The weight loss of 3.21% from room temperature to 233 °C was caused by the evaporation of absorbed moisture in the powder[1]. Weight loss of 21.33% from 262 °C to 546 °C was caused by the burning of amorphous carbon and the loss of 21.32% between 571 °C and 787 °C was assigned to the combustion of the crystallized MWCNTs in the sample[2].

Table S1 Atomic percentage calculated by processing the fitted Nb3d, C1s and O1s XPS spectra.

**Nb3d**					
**Component**	$Nb^{5+}3d_{2/5}$		$Nb^{5+}3d_{3/2}$		
**Atomic amount (At%)**	59.79		40.21		
**C1s**					
**Component**	C-C	C-O	C=O	O-C=O	π-π
**Atomic amount (At%)**	59.58	27.74	8.31	5.84	1.53
**O1s**					
**Component**	Nb-O	C=O	C-O		
**Atomic Amount (At%)**	79.19	14.67	6.14		

The atomic amount of different component were calculated by processing the XPS survey and fitted XPS spectra using CasaXPS.

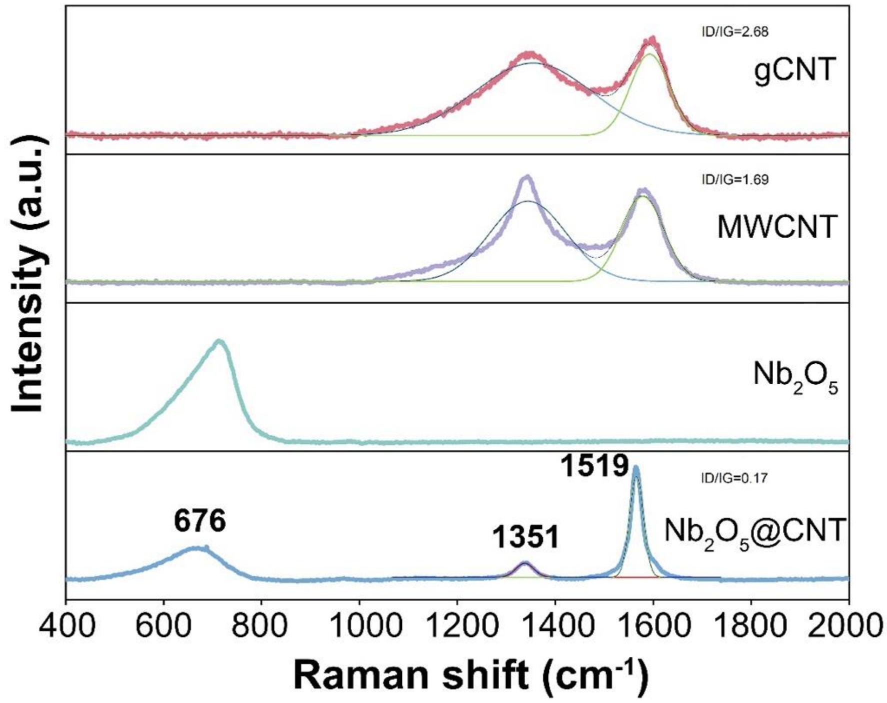

Figure S3 Raman spectra of Nb_2O_5@CNT, Nb_2O_5, MWCNT and gCNT.

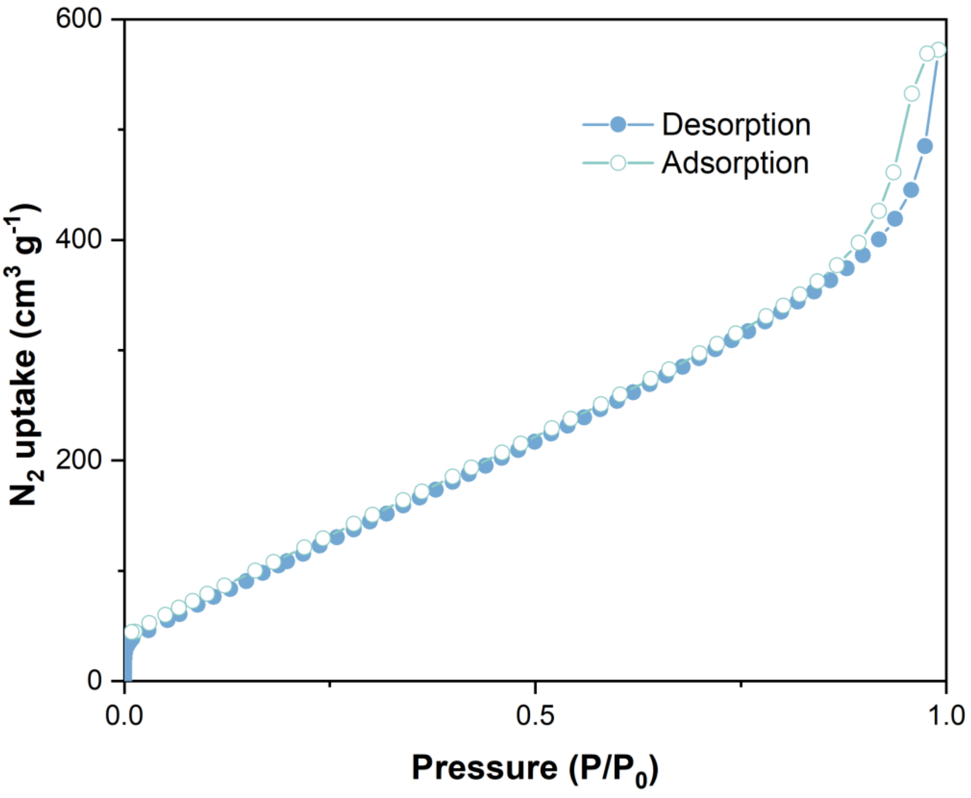

Figure S4 N2 adsorption/desorption isotherm of Nb_2O_5@CNT.

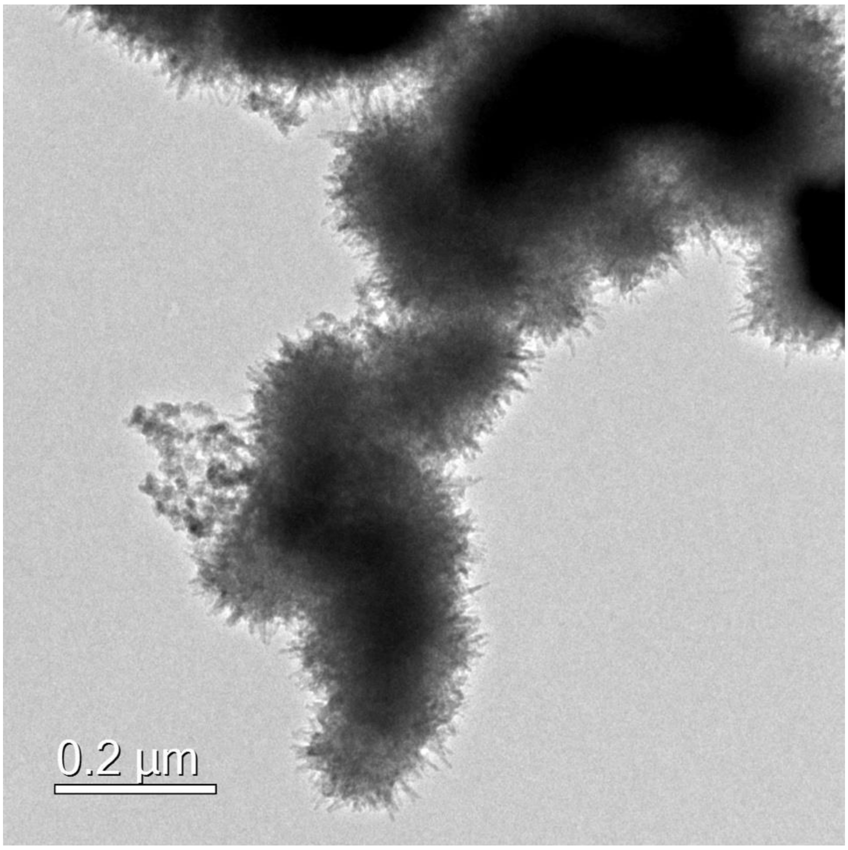

Figure S5 TEM image of the Nb_2O_5@CNT composite.

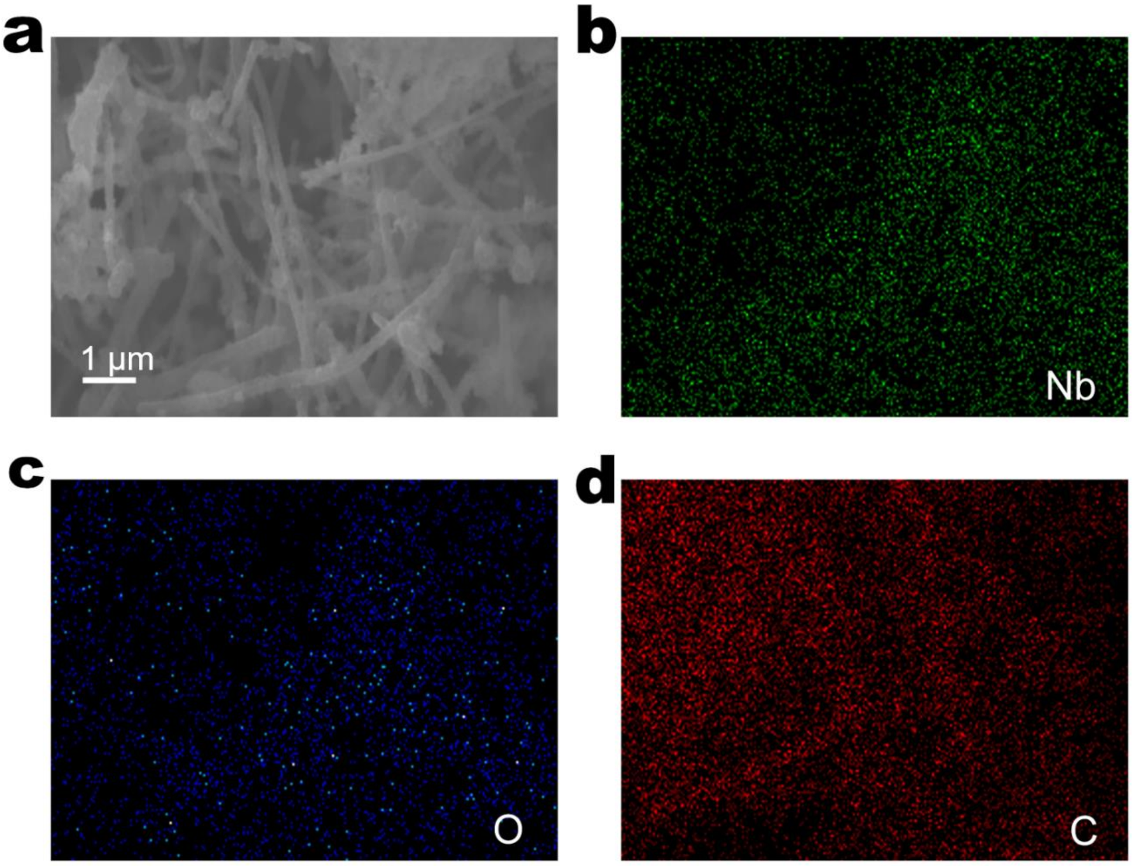

Figure S6 EDS profile of the Nb_2O_5@CNT composite.

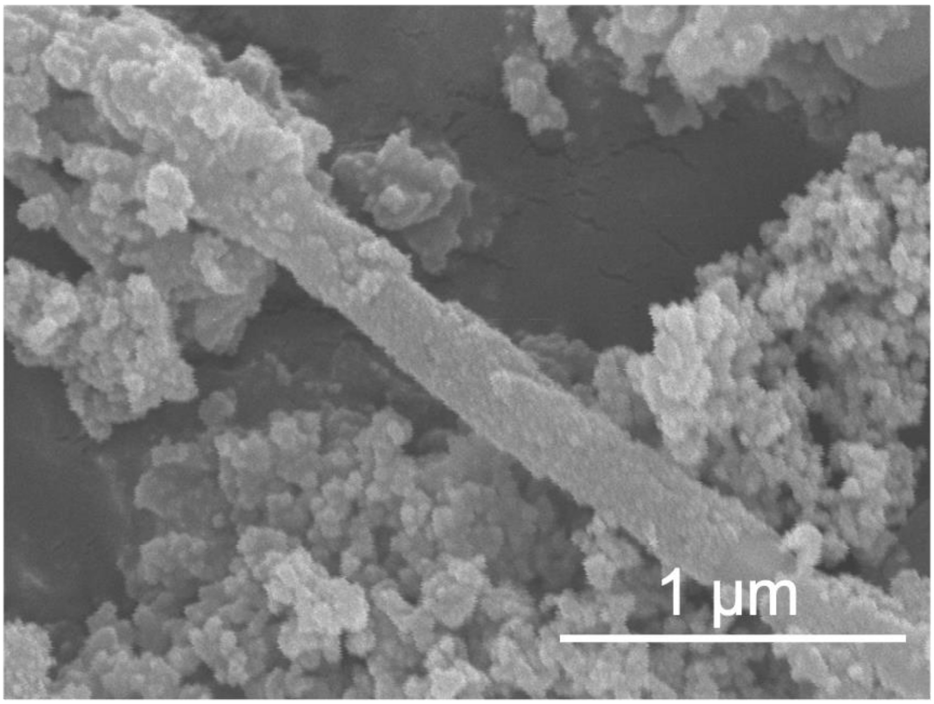

Figure S 7 SEM image of Nb_2O_5@0.5CNT (low carbon content, synthesised by halving the amount of carbon precursors).

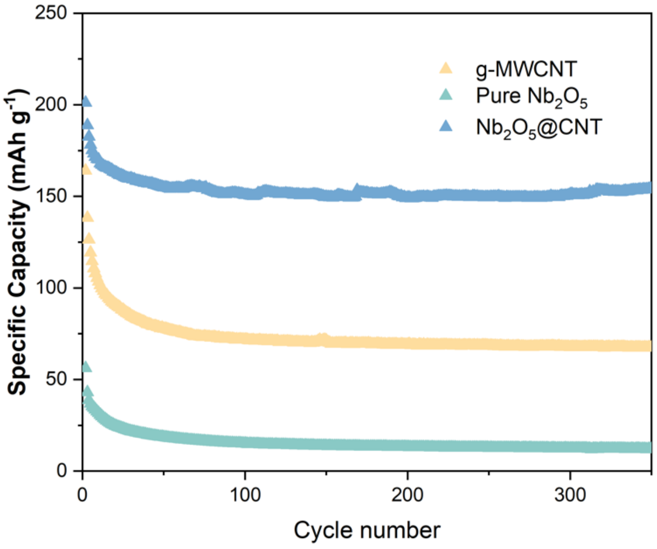

Figure S8 CD profile of Nb_2O_5@CNT (0.94 mg cm-^{2}), g-MWCNT (0.81 mg cm^{-2}) and Nb_2O_5.(0.96 mg cm^{-2}) at 0.2 A g^{-1}.

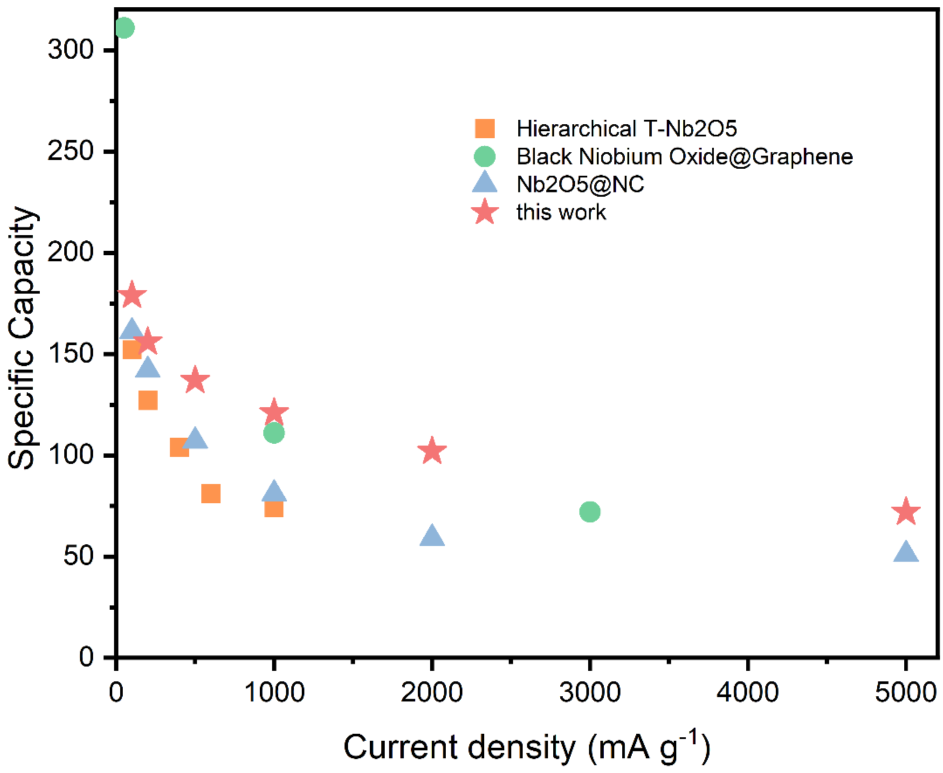

Figure S9 Comparison of Nb_2O_5@CNT presented in this paper with other Nb_2O_5-based PIB anode in published works.

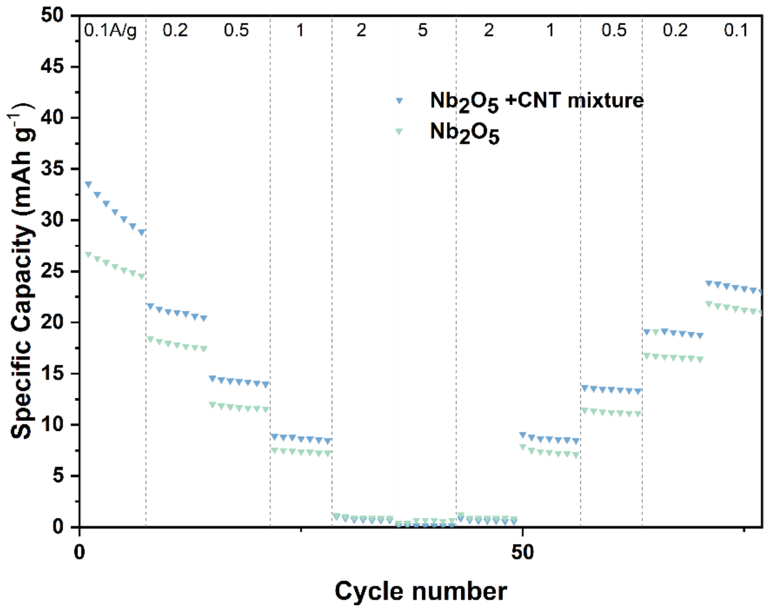

Figure S10 Rate performance of Nb_2O_5 (0.96 mg cm^{-2}) and Nb_2O_5+CNT (1.06 mg cm^{-2}) mixture at current density of 0.1, 0.2, 0.5, 1, 2, 5 A g^{-1}.

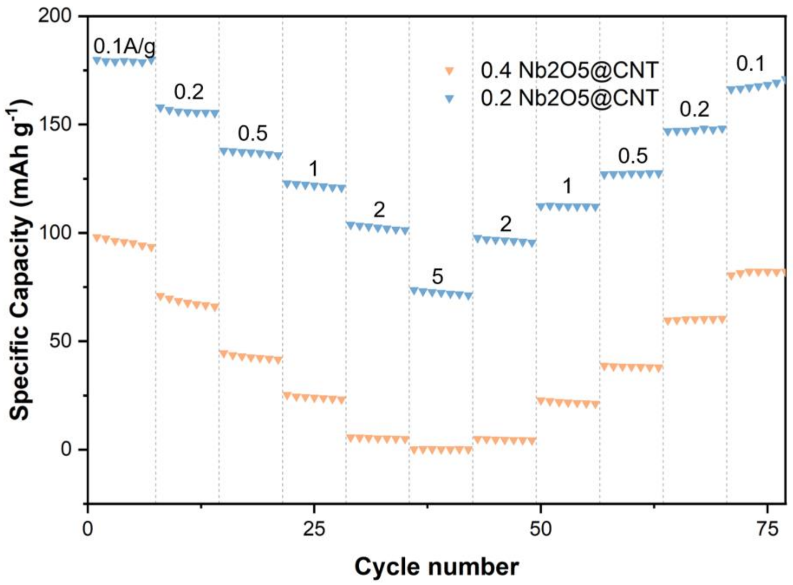

Figure S11 Rate performance of $0.4Nb_2O_5$@CNT (low carbon content) and $0.2Nb_2O_5$@CNT (high carbon content) in KIBs.

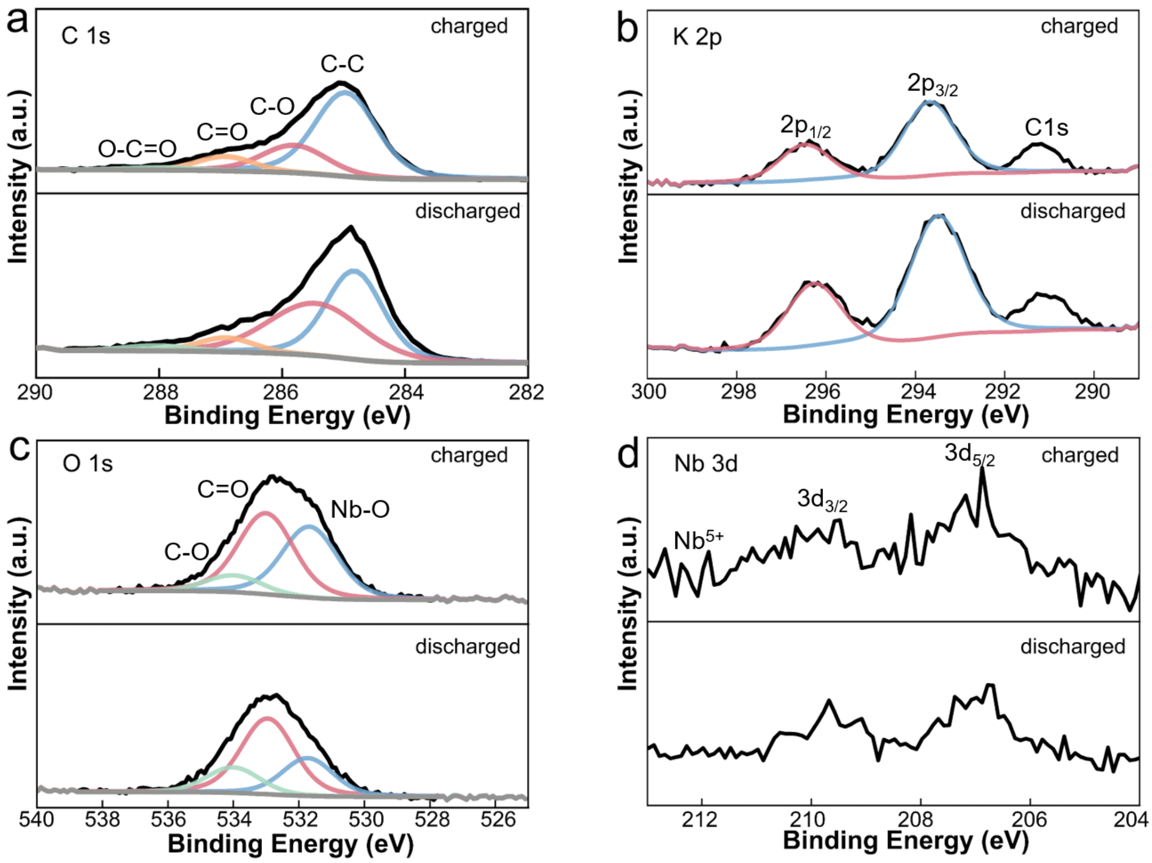

Figure S 12 The XPS fitting curves of (a) C 1s; (b) K 2p; (c) O 1s and (d) Nb 3d for cycled Nb_2O_5@CNT at different (dis)charge states.
